# Evaluating the Hydrologic Performance of Low Impact Development Scenarios in a Micro Urban Catchment

**DOI:** 10.3390/ijerph15020273

**Published:** 2018-02-05

**Authors:** Chunlin Li, Miao Liu, Yuanman Hu, Rongqing Han, Tuo Shi, Xiuqi Qu, Yilin Wu

**Affiliations:** 1 CAS Key Laboratory of Forest Ecology and Management, Institute of Applied Ecology, Chinese Academy of Sciences, Shenyang 110016, China; lichunlin@iae.ac.cn (C.L.); huym@iae.ac.cn (Y.H.); tuoshi0411@163.com (T.S.); quqi19951123@163.com (X.Q.); 2 School of Geography and Environment, Shandong Normal University, Jinan 250358, China; hrqsd@126.com (R.H.); wyl19900828@163.com (Y.W.); 3 College of Resources and Environment, University of Chinese Academy of Sciences, Beijing 100049, China

**Keywords:** rainfall-runoff, cost-effectiveness curve, SUSTAIN, Urban non-point pollution, urbanization

## Abstract

As urbanization progresses, increasingly impervious surfaces have changed the hydrological processes in cities and resulted in a major challenge for urban stormwater control. This study uses the urban stormwater model to evaluate the performance and costs of low impact development (LID) scenarios in a micro urban catchment. Rainfall-runoff data of three rainfall events were used for model calibration and validation. The pre-developed (PreDev) scenario, post-developed (PostDev) scenario, and three LID scenarios were used to evaluate the hydrologic performance of LID measures. Using reduction in annual runoff as the goal, the best solutions for each LID scenario were selected using cost-effectiveness curves. The simulation results indicated that the three designed LID scenarios could effectively reduce annual runoff volumes and pollutant loads compared with the PostDev scenario. The most effective scenario (MaxPerf) reduced annual runoff by 53.4%, followed by the sponge city (SpoPerf, 51.5%) and economy scenarios (EcoPerf, 43.1%). The runoff control efficiency of the MaxPerf and SpoPerf scenarios increased by 23.9% and 19.5%, respectively, when compared with the EcoPerf scenario; however, the costs increased by 104% and 83.6%. The reduction rates of four pollutants (TSS, TN, TP, and COD) under the MaxPerf scenario were 59.8–61.1%, followed by SpoPerf (53.9–58.3%) and EcoPerf (42.3–45.4%), and the costs of the three scenarios were 3.74, 3.47 and 1.83 million yuan, respectively. These results can provide guidance to urban stormwater managers in future urban planning to improve urban water security.

## 1. Introduction

Rapid urbanization increases the area of impervious surfaces, a major factor that affects urban hydrologic processes through increased surface runoff volume, increased runoff velocity, decreased time of concentration, and decreased groundwater recharge [1,2,3,4]. Human activities in cities have been shown to increase non-point source pollution of contaminants such as oils, greases, metals, and pesticides into streams and rivers during rainfall events [5,6,7,8]. Pollution from human activity produces waste water and storm water quality that can be detrimental to human health and to aquatic organisms [9,10]. In order to mitigate the negative impacts of urbanization, many stormwater management strategies have been developed, such as best management practices (BMPs), low impact development (LID), green infrastructure (GI), and water-sensitive urban design (WSUD) [11,12]. China has put forward a new urban storm water management concept based on LID known as “Sponge City” to address the negative effects of urbanization on water systems [13].

LID is a stormwater management approach that seeks to preserve the pre-development hydrology of a given site using decentralized, micro-scale control measures [14]. There are many kinds of LID measures, including bioretention, grass swales and channels, green rooftops, and rain barrels [15,16]. Zare et al. (2012) used the storm water management model (SWMM) with The Non-dominated Sorting Genetic Algorithm-II(NSGA-II) to control both the quality and quantity of urban runoff [17]. Kong et al. (2017) explored the hydrological responses of stormwater runoff characteristics to four different land use conversion scenarios at the city scale, and the simulation results corroborated the effectiveness of LID controls and design in providing some flood reduction benefits [18]. Different LID measures require different applicable principles [19]. To implement LID measures, appropriate LID types, sizes, and spatial locations should be designed with the LID applicable principles and the overall construction of the development site [20,21]. The LID design should fully consider the local geology, hydrology, soil, underlying surface, land use types, and other conditions. 

In stormwater management practice, models are often used to assess the runoff and pollution reduction of LIDs, including the SCS (Soil Conservation Service), SWAT (Soil-Water Assessment Tool), MOUSE (Model for Urban Sewers, Danish Hydraulic Institute, 1995), Hydro CAD, SWMM, and the system for urban stormwater treatment and analysis integration (SUSTAIN) [10,14,22,23,24]. SUSTAIN model developed by the US EPA is an important hydrological model for evaluating the performance and cost of BMPs and LIDs [25,26]. Mao et al. (2017) used SUSTAIN to assess the reduction of flow volume and pollutant loads of aggregate LID-BMPs in Foshan New City, China [27]. Chen et al. (2014) analyzed the SS reduction of different LID types, and found the LID area ratio affected the pollution reduction performance [26].

Most previous studies focused on modelling and analyzing the quality and quantity reduction of LID scenarios, but few studies focused on the cost of LIDs. In SUSTAIN, there is an optimization programming module that can be used to optimize the distribution of different LID types to get the minimum cost. The study has several goals: (1) to calibrate and validate the SUSTAIN model; (2) to optimize the configuration of the LID scenarios; (3) to evaluate the performance of the designed LID scenarios on runoff volume and pollutant loads reduction.

## 2. Materials and Methods

### 2.1. Study Area and Data

**Figure 1:**
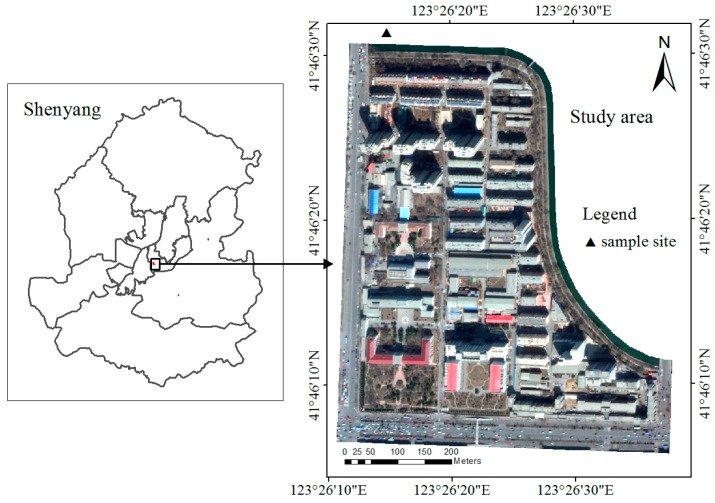
Study area and sample site.

The study catchment area was a residential–educational mixed block in the city of Shenyang, which is the largest industrial city in Northeastern China (Figure 1). Shenyang has a temperate continental monsoon climate, with a mean annual precipitation of 510–680 mm, most of which falls from June to August. The case study catchment area comprises high-density residential (56.1%) and educational (43.9%) areas and is 24.2 hm^2^. Impervious surfaces cover 69.3% of the catchment, and gently slope from southeast to northwest with an average slope of 0.1%. The sewer system is separated and constructed of concrete pipes, and stormwater runoff flows past conduits to the final outfall at the north of the study area. 

Rainfall data was monitored using a tipping bucket rain gauge that could accurately measure rainfall as low as 0.25 mm (0.01 in). The rain gauge was located on a grassy area of the catchment. Rainfall ranged from 4.6 to 33.8 mm, and the average rainfall intensity ranged from 1.3 to 12.1 mm/h. The antecedent dry weather periods ranged from 5 to 10 days (Table 1). Runoff samples were collected manually in polyethylene bottles during three rainfall events from July to August 2012. The samples were treated and analyzed in the laboratory within 24 h of collection. All storm runoff samples were analyzed for total suspended solids (TSS), chemical oxygen demand (COD), total nitrogen (TN), and total phosphorus (TP) using standard methods [28]. The pollutions we selected were ubiquitous in the environment and also formed during natural processes and human activities, including motor vehicle emissions, coal burning, tire wear, asphalt leaching, and runoff from different urban surfaces [29,30]. Additionally, the four pollutions are classified as priority pollutants for stormwater [31]. Runoff volume and pollutant concentrations of the three rainfall events were used for calibrating and validating the SUSTAIN model.

### 2.2. Model Description

The SUSTAIN model was developed by the US EPA to assist in developing implementation plans for flow and pollution control to protect source waters and meet water quality goals [32]. The SUSTAIN model is useful for selecting the LID types, setting LID location, and evaluating the hydrological performance. It is built on a base platform interface using ArcGIS, enabling users to easily define input files and modify parameters [27]. The required data for SUSTAIN include various types of digital maps and its attribute data, meteorological data, hydrological data, and cost data of different LID, etc. Over ten types of LID/BMP facilities can be chosen and placed in appropriate sites based on suitability criteria including elevation, slope, soil type, urban land use, roads, stream location, and drainage area. The cost module estimates the overall costs of implementing LID based on fundamental construction components [33]. SUSTAIN has an optimization programming module that can be used to optimize the location, distribution, and cost of GI based on the cost-effectiveness curve. The optimization module and post-processor is useful for assessing different LID designs in terms of cost and performance, and selecting the most cost-effective plan [26]. Taking the characteristics of the study catchment and the distribution of manholes into consideration, the catchment was divided into 38 subcatchments and 34 junctions, representing a detailed discretization. In SUSTAIN, the model provides Green Ampt and Horton for simulating infiltration, and both of these methods are widely used to simulate rainfall infiltration processes [25,26,32]. In this paper, Horton’s equation was used for infiltration, and flow routing computations were based on the dynamic wave theory.

### 2.3. Goodness-of-Fit Test

Calibration and validation of the hydrological model required that the dominant parameters of each conceptual component be determined so the outputs could present an accurate response of the catchment [34]. SUSTAIN was calibrated and validated for runoff quantity and quality simulation before the LID scenarios evaluation. Model performance evaluation was assessed with Nash–Sutcliffe coefficient (*R_NS_*), relative error (*RE*), and coefficient of determination (*R^2^*), as shown in Equations (1)–(3), respectively [35].

(1) $$R_{NS}=1-\frac{\sum_{t=1}^{n}{\left(q_{t}^{obs}-q_{t}^{sim}\right)}^{2}}{\sum_{t=1}^{n}{\left(q_{t}^{obs}-\bar{q_{t}^{obs}}\right)}^{2}},$$

(2) $$RE=\frac{\sum_{t=1}^{n}\left|q_{t}^{obs}-q_{t}^{sim}\right|}{\sum_{t=1}^{n}q_{t}^{obs}}\times 100,\;\mathrm{and}$$

(3) $$R^{2}={\left(\frac{\sum_{t=1}^{n}\left(q_{t}^{obs}-\bar{q_{t}^{obs}}\right)\left(q_{t}^{sim}-\bar{q_{t}^{sim}}\right)}{\sqrt{\sum_{t=1}^{n}{\left(q_{t}^{obs}-\bar{q_{t}^{obs}}\right)}^{2}}\sqrt{\sum_{t=1}^{n}{\left(q_{t}^{sim}-\bar{q_{t}^{sim}}\right)}^{2}}}\right)}^{2},$$

in which $q_{t}^{obs}$ (m^3^) is the observed flow at time *t*; $q_{t}^{sim}$ (m^3^) is the predicted flow at time *t*; $\bar{q_{t}^{obs}}$ (m^3^) is the average observed discharge; $\bar{q_{t}^{sim}}$ (m^3^) is the average predicted discharge; *t* is time, and *n* is the total number of time steps.

*R_NS_* measures the goodness of fit by comparing both the volume and shape of the discharge profile [36]. It also provides a comparison between the efficiency of the chosen model and a description of the data as the mean of the observations [37]. The optimal simulation value occurs when *R_NS_* is close to 1. *RE* is the error percentage (the ratio of absolute error and the observed values) that shows the reliability of the predicted value. *R*^2^ indicates how well the observed values are replicated by the simulation, as a proportion of the total variation of the outcomes explained by the simulation [35].

### 2.4. Designed Scenarios

In this study, we attempted to minimize the impervious area and take full advantage of the infiltration capacity to reduce annual runoff volume while considering the local environment and LID construction cost. We selected appropriate LID measures (rain barrel, bioretention, porous pavement, and green roof) based on the condition analysis of study area. Buildings with flat roofs could have green roofs, and buildings with sloping roofs could have rain barrels. For parking lots and impervious roads (except main roads) porous pavement could be implemented, and existing green gardens along roadways could be converted to bioretention. For comparison, five scenarios were set in SUSTAIN: pre-development (PreDev scenario), post-development (PostDev scenario), maximized LIDs scenario (MaxPerf scenario), economic LIDs scenario (EcoPerf scenario), and sponge city scenario (SpoPerf scenario).

(1) PreDev scenario

This scenario assumed the catchment has the same conditions as before development. Therefore, it was represented by using pre-development land-use type, which is green space.

(2) PostDev scenario

This scenario represented the existing conditions of the residential–educational mixed block. This is also representative of most of Shenyang’s communities, which have not implemented LID projects. It is the baseline for assessing the performance of the LID measures.

(3) MaxPerf scenario

**Figure 2:**
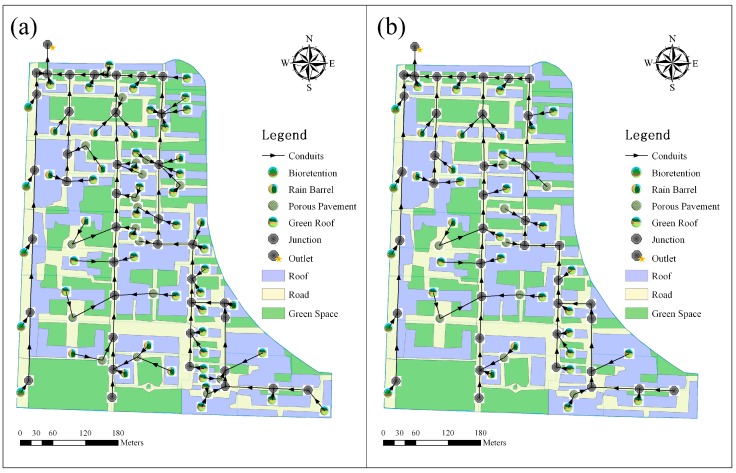
LIDs layout of three LID scenarios: (**a**) is the LIDs layout under MaxPerf and SpoPerf scenario; (**b**) is the LIDs layout under EcoPerf scenario.

Under this scenario, the primary consideration was to maximize the LID’s effectiveness. Four types of LIDs (rain barrel, bioretention, porous pavement, and green roof) were placed in all suitable locations according to the actual conditions, to enhance stormwater control performance. The MaxPerf scenario included 13 rain barrel sites, five bioretention sites, 18 porous pavement sites, and 35 green roof sites (Figure 2a).

(4) EcoPerf scenario

The costs and performance of LIDs were both taken into consideration under this scenario. The LID measures were mainly arranged in the areas where the impervious surface was concentrated. The EcoPerf scenario included eight rain barrel sites, five bioretention sites, nine porous pavement sites, and 22 green roof sites (Figure 2b).

(5) SpoPerf scenario

China has put forward the Sponge City urban storm water management strategy to address the negative effects of urbanization of water environments [13,38,39]. According to the requirements of Sponge City construction, the target annual rainfall runoff control rate in Shenyang is 75–85%. Therefore, the layout of LIDs was the same as the MaxPerf scenario (Figure 2a), and we wanted to find the most cost-effective solution to meet the requirements of Sponge City through the optimization module of SUSTAIN.

Actual daily rainfall data of 2015 were used to simulate runoff and pollutant loads for all scenarios. The costs of various LID measures were set according to the local price level and the Technical Guide for Sponge City Construction (TGSCU) [40]. In this study, we only consider the construction cost of the LID measures and do not include the maintenance costs. The cost data used for different LIDs in Shenyang is summarized in Table 2.

## 3. Results

### 3.1. Calibration and Verification

**Figure 3:**
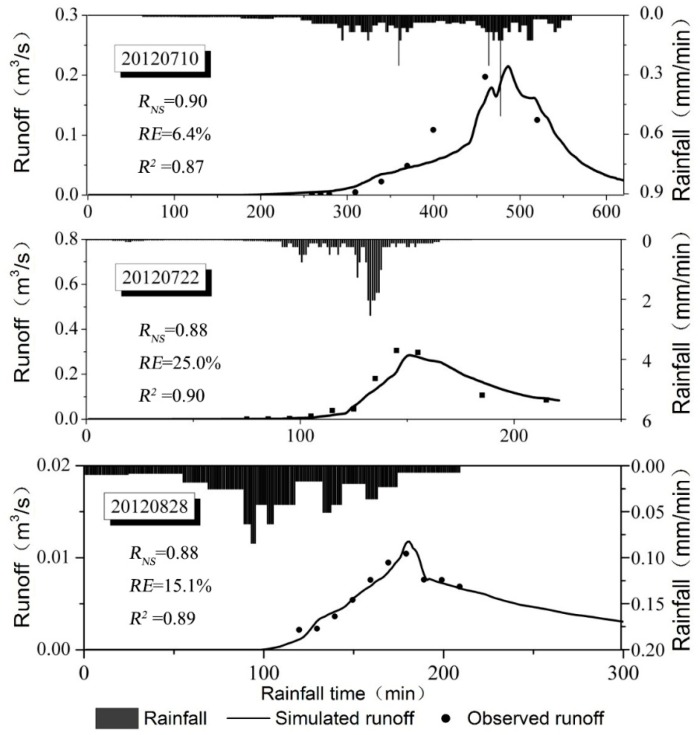
Comparison between the simulated and observed values in SUSTAIN for three rainfall events. *R_NS_* is Nash–Sutcliffe coefficient; *RE* is relative error, and *R*^2^ is coefficient of determination.

Before applying the SUSTAIN model to assess the performance of LID scenarios, the model was calibrated and verified using monitored runoff volume and pollutant data of three rainfall events in 2012. Of these rainfall events, the 2nd and 3rd rainfall events were used for calibration, and the 1st rainfall event was used to validate the model. The observed values and simulated results were compared, and the goodness of fit assessed by calculating the *R_NS_*, *RE*, and *R*^2^. The simulated flow results and observed runoff are shown in Figure 3. The simulated runoff curves were well fit for observed volume points. The *R_NS_* and *R*^2^ of the three events were 0.9, 0.88, 0.88, and 0.87, 0.90, 0.89, respectively. The *RE* for the 1st and 3rd events were small, 6.4% and 15.1%, and for the 2nd event slightly larger at 25.0%. For water quality simulations, the goodness-of-fit test results of four pollutants are summarized in Table 3. The calibration and verification results of runoff quality were not as robust as those for runoff volume. However, the *R_NE_* of four pollutants were concentrated at approximately 0.8 (0.69–0.97), the *RE* were less than 27%, and the *R*^2^ were more than 0.74. The calibration and verification results indicated that the model structure and parameters matched the runoff-producing pattern and the calibrated SUSTAIN was suitable for simulating LID scenarios in the study area.

### 3.2. LID Scenarios Optimization

**Figure 4:**
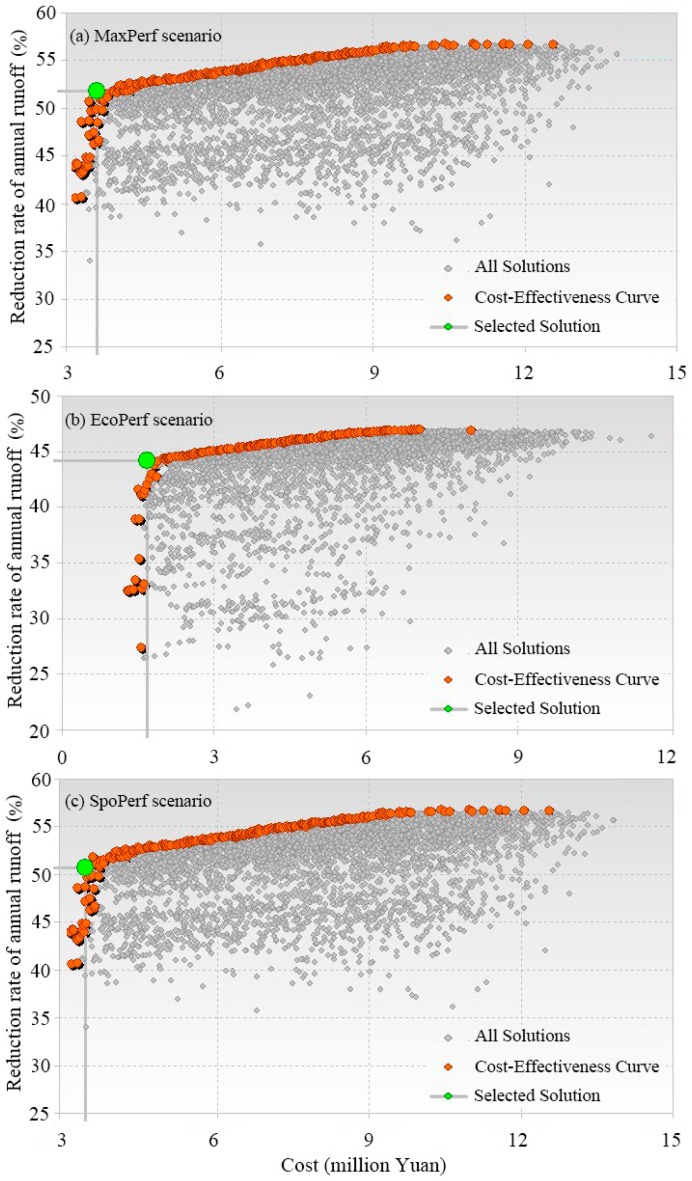
Cost-effectiveness analysis of different scenarios. The red points represent the cost-effective solutions. The green point is the selected best solution for each scenario. (**a**), (**b**), and (**c**) are the cost-effectiveness curves of MaxPerf scenario, EcoPerf scenario, and SpoPerf scenario, respectively.

With the goal of reducing annual runoff, the optimization module of SUSTAIN was used to optimize the configuration of different types of LIDs to find the optimal combination of LID scenarios. The decision variables were the unit amount of different LID measures. Each subcatchment has a unique drainage area; thus, the range and increment were set separately. The NSGA-II is one of the promising MOEAs and has been successfully applied in many engineering fields. It was developed to address issues of computational complexity, as well as to provide an explicit mechanism for diversity preservation by using the non-dominated sorting and ranking selection with the crowded comparison operator [17]. NSGA-II was integrated in the optimization module of SUSTIAN, and it is a useful tool to simulate the appropriate LIDs [41,42]. Based on the calibrated model and precipitation data of 2015, five thousand iterations were conducted for three scenarios, and the resulting cost-effectiveness curves are illustrated in Figure 4. 

In the cost-effectiveness curve, the X axis represents the total cost, and Y axis represents the annual runoff volume reduction rate of LIDs compared to the PostDev scenario. Each point in the figure represents a possible solution in the optimization process. The red points represent the cost-effective solutions. The best solution is the “knee-of-curve” point and the green point is the selected solution for each scenario.

For the MaxPerf scenario, the optimized solution was chosen at the “knee-of-curve” point, which had a 51.8% total runoff volume reduction rate compared to the PostDev scenario (Figure 4), and a total cost of 3.74 million Yuan (Table 4). For the EcoPerf scenario, the optimized solution had a 44.2% total runoff volume reduction rate, and a total cost of 1.83 million Yuan. Compared with the EcoPerf scenario, the runoff reduction rate of the MaxPerf scenario increased by 7.6%, while the cost increased by 1.91 million Yuan (104%). According to the Sponge City urban policy, the average annual rainfall runoff control rate of Shenyang should above 75%. These requirements can be met by reducing 51% runoff volume compared to the PostDev scenario using LID treatments based on the consideration of rainfall and the current conditions in the study area. The SpoPerf scenario was selected as the solution of 51% runoff volume reduction rate from the cost-effectiveness curve of the MaxPerf scenario, at a total cost of 3.47 million Yuan. The cost distribution of the four LID components for each scenario is shown in Table 4. In the selected best solutions for each scenario, green roofs accounted for the most cost, followed by porous pavement, bioretention, and rain barrels.

### 3.3. Performance of Scenarios

**Figure 5:**
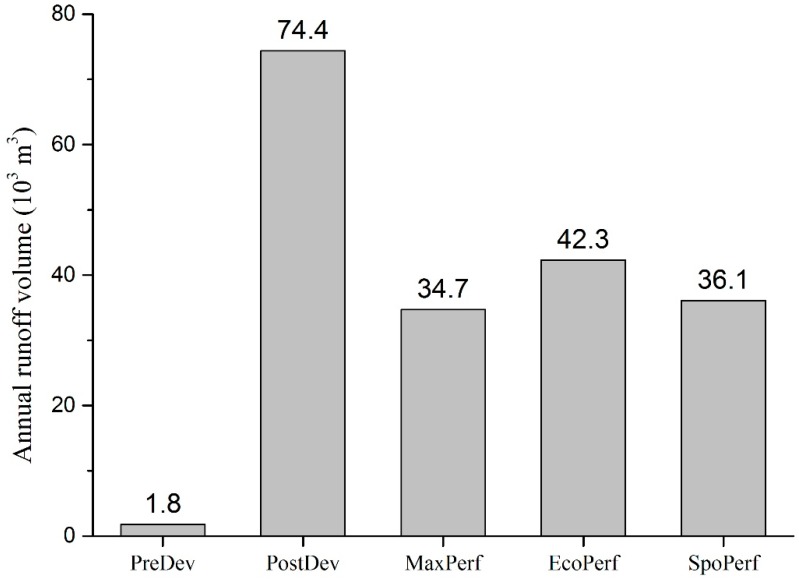
Annual runoff volume of different scenarios.

The performances of five scenarios described above were simulated using SUSTAIN. For the present study, the target indexes for evaluating system performance were total annual runoff volume and pollutant loads. The results showed that the annual runoff volume of PostDev scenarios increased by 72.6 million m^3^ (40 times) when compared to the PreDev scenario, clearly indicating the negative impacts of urbanization (Figure 5). The three designed LID scenarios could effectively reduce approximately half of the annual runoff. The most effective scenario is MaxPerf, which reduced 53.4% of annual runoff of the PostDev scenario, followed by the SpoPerf (51.5%) and EcoPerf scenario (43.1%). However, these three LID scenarios could not reduce annual runoff to the same level as before development (PreDev scenario). The runoff control efficiency of the MaxPerf and SpoPerf scenarios increased by 23.9% and 19.5% compared with the EcoPerf scenario; however, the costs increased by 104.4% and 83.6%, respectively (Table 4).

**Figure 6:**
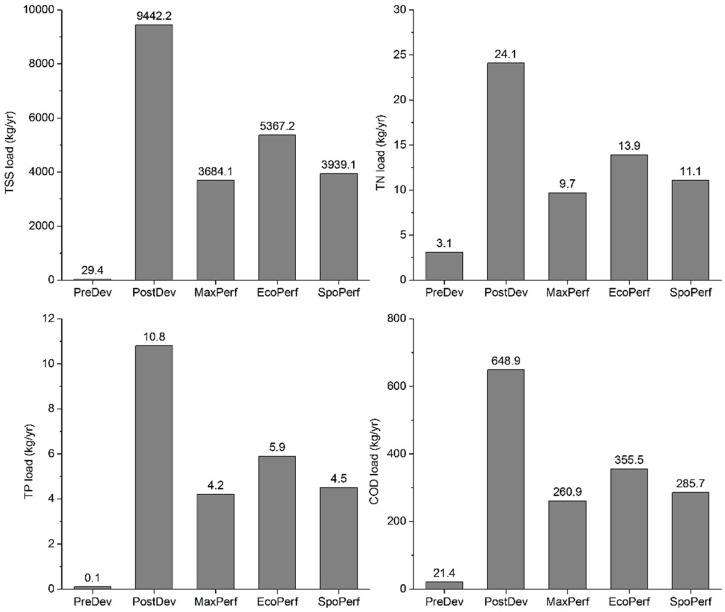
Pollutant loads of different scenarios.

In this study, reducing annual runoff volume is the goal of LID optimization, but the discharge of non-point source pollutants is also reduced as runoff is reduced. Concentrations of TSS, TN, TP, and COD in runoff increased from the PreDev to the PostDev scenario (Figure 6). However, the simulation results demonstrated that by properly designing and implementing LID measures for the study catchment, significant reductions in non-point pollution could be achieved. For pollutant load, the reduction rate of four pollutants under the MaxPerf scenario was 59.8–61.1%, followed by the SpoPerf scenario (53.9–58.3%) and the EcoPerf scenario (42.3–45.4%), and the costs of the three scenarios were 3.74, 3.47, and 1.83 million Yuan, respectively. For all three LID scenarios, TP had the largest reduction rate, and TN had the smallest reduction rate.

## 4. Discussion

### 4.1. Hydrologic Performance of LID in Micro Urban Catchments

The SUSTAIN model is a useful and efficient tool for LID evaluation and optimization, as demonstrated by the current study and many previous studies [27,33,43,44,45]. The simulation results indicated that LID measures can have an enormous impact on the rainfall-runoff process. Compared with the PostDev scenario, the three LID scenarios could reduce annual runoff by 43.1–53.4%, and reduce four non-point pollutant loads by 42.3–61.1%. Despite significant reductions after LID treatments, runoff volume and pollutant loads were still far above those of the PreDev scenario. This may be related to the type of LID measures selected in this study. Bioretention and wetland ponds may offer more efficient runoff and pollution control effects [46,47]. In addition, aggregate LID can treat stormwater through multilevel LID measures compared with individual LID, and more runoff and pollution can be reduced [27,48]. We found that as the LID cost increased dramatically from the EcoPerf scenario to the MaxPerf scenario, the coinciding reductions in runoff and pollutants were not as great. Therefore, optimization using models and scenarios prior to LID construction is a valuable tool to control costs. 

### 4.2. Applicability and Implications

As rapid urbanization occurs in China, controlling annual runoff volume and non-point pollution is the most important goal of urban stormwater management [11,30,49,50]. Financial risks and health threats attributed to urban runoff have always been challenging issues in urban planning of large cities. Many previous studies have shown that LID can significantly reduce non-point source pollution. Gao et al. (2015) indicated that BMP plan would reduce TSS, Zn, TN, TP, and COD by 62%, 55%, 57%, 55%, and 60% [25]. Jia et al. (2015) found that pollution removal efficiency of the LID-BMP treatment for COD, NH_3_-N, TN, TSS, and TP is 18.52%, 73.48%, 74.00%, 34.85%, and 95.26% [51]. The pollution reduction rate of four pollutants under the MaxPerf scenario was 59.8–61.1%. The effective removal of pollution from urban rainfall runoff by LID measures can reduce the negative impact of runoff on human health. In this study, we only used annual runoff reduction as the simulation goal to optimize the LID types and amounts. In addition, pollutant reduction can also be used as an optimization goal and provide insight to urban managers for non-point source control. Using the cost-effectiveness curve, urban governments could choose the most efficient LID plan based on available funding. In addition, the analysis of performance and cost of LID scenarios could provide a viable approach to promote the LID constructions of Sponge City and stormwater management. 

### 4.3. Limitations and Uncertainties

Our research is limited, as it is based on model simulations rather than monitored data. In this study, three real rainfall events were used to calibrate and verify stormwater and pollutant runoff in the SUSTAIN model. Although simulated runoff curves fit well with the observed data, the limited number of monitored events could result in lower accuracy of the calibrated parameters for a wide range of storm conditions. The simulation results of the LID scenarios cannot be calibrated, because they are all designed plans, for which the reduction results can only be used as a design reference of LID reconstruction in this catchment. Currently, there are various individual LID measures, such as bioretention areas, grass filter strips, and permeable pavement. Different measures can control water quantity and quality through one or more processes (e.g., interception, absorption, degradation, infiltration) according to their design parameters [52,53,54,55]. Some previous studies indicated that combined LID measures have better hydrological improvements compared with individual LID measures [56,57]. Mao (2017) reported that 40% of flow volume and over 60% of all pollutant loads were reduced by aggregate LIDs [27]. However, in the current study, only four common individual LID measures were investigated. In addition, only the amount (not the parameters) of LID measures was changed in the optimization process. Thus, to better understanding the performance of LID measures, full-ranged investigations and simulated analyses are needed.

## 5. Conclusions

In this study, we used SUSTAIN to simulate and evaluate the hydrologic performance of LID scenarios in a micro urban catchment. The SUSTAIN model was calibrated and validated by data from three observed rainfall-runoff events. Three LID scenarios (MaxPerf, EcoPerf, and SpoPerf) were designed and presented in the model. The reduction of runoff volume and pollution loads of LID scenarios were assessed and compared with the situation before and after development (PreDev and PostDev scenario).

The simulation results indicated that the LID measures have an enormous impact on the rainfall-runoff process. Compared with the PostDev scenario, the three LID scenarios could reduce annual runoff by 43.1–53.4%. The reduction rates of four pollutants (TSS, TN, TP, and COD) under the MaxPerf scenario were 59.8–61.1%, followed by SpoPerf (53.9–58.3%) and EcoPerf (42.3–45.4%), and the costs of the three scenarios were 3.74, 3.47, and 1.83 million yuan, respectively. However, as the LID cost increased dramatically from EcoPerf scenario to MaxPerf scenario, the reduction in stormwater and pollutant runoff did not significantly improve. The present study developed a method to evaluate the performance of designed LID scenarios on runoff and pollutant reduction, and optimized the configuration of LID scenarios to reduce costs. 

## Figures and Tables

**Table 1:** Characteristics of rainfall events.

Date (d-m-y)	Rainfall (mm)	Duration (h)	Average Intensity (mm/h)	Max Rainfall Intensity (mm/h)	Antecedent dry Weather Period (day)
10 July 2012	16.0	6.7	2.4	5.0	5
22 July 2012	33.8	2.8	12.1	28.7	10
28 August 2012	4.6	3.5	1.3	2.4	9

**Table 2:** Cost data for different LIDs in Shenyang.

LIDs	Cost Rang in TGSCU	Total Cost in Shenyang
Bioretention (Yuan/m^2^)	150–800	500
Rain Barrel (Yuan/unit)	50–150	110
Porous Pavement (Yuan/m^2^)	60–200	130
Green Roof (Yuan/m^2^)	100–300	200

**Table 3:** Goodness-of-fit test results of pollutants for model calibration and verification.

Pollutants	Events	Goodness of Fit Indicators		
		*R_NS_*	*RE* (%)	*R*^2^
TSS	20120710	0.88	22.4	0.90
	20120722	0.96	12.1	0.74
	20120828	0.75	11.8	0.75
TP	20120710	0.91	22.7	0.96
	20120722	0.79	27.0	0.95
	20120828	0.78	11.4	0.86
TN	20120710	0.97	17.1	0.97
	20120722	0.84	13.5	0.89
	20120828	0.69	15.0	0.89
COD	20120710	0.95	8.4	0.96
	20120722	0.86	14.4	0.90
	20120828	0.83	11.2	0.86

**Table 4:** Optimized scenarios and their costs.

Scenarios		Bioretention (m^2^/million Yuan)	Rain Barrel (barrel/million Yuan)	Porous Pavement (m^2^/million Yuan)	Green Roof (m^2^/million Yuan)	Total (million Yuan)
MaxPerf	Units	951	97	9772	9891	-
	Cost	0.48	0.01	1.27	1.98	3.74
EcoPerf	Units	314	169	5619	4593	-
	Cost	0.16	0.02	0.73	0.92	1.83
SpoPerf	Units	866	97	9816	8749	-
	Cost	0.43	0.01	1.28	1.75	3.47
